# Protein lysine methyltransferase SMYD3 is involved in tumorigenesis through regulation of HER2 homodimerization

**DOI:** 10.1002/cam4.1099

**Published:** 2017-06-22

**Authors:** Yuichiro Yoshioka, Takehiro Suzuki, Yo Matsuo, Giichiro Tsurita, Toshiaki Watanabe, Naoshi Dohmae, Yusuke Nakamura, Ryuji Hamamoto

**Affiliations:** ^1^ Section of Hematology/Oncology Department of Medicine The University of Chicago 5841 S. Maryland Ave, MC2115 Chicago Illinois 60637; ^2^ Department of Surgical Oncology Graduate School of Medicine The University of Tokyo 7‐3‐1 Hongo Bunkyo‐ku Tokyo 113‐8654 Japan; ^3^ Biomolecular Characterization Unit RIKEN Center for Sustainable Resource Science 2‐1 Hirosawa Wako Saitama 351‐0198 Japan; ^4^ OncoTherapy Science Inc. 3‐2‐1 Sakado, Takatsu‐ku Kawasaki Kanagawa 213‐0012 Japan; ^5^ Department of Surgery IMSUT Hospital Institute of Medical Science The University of Tokyo 4‐6‐1 Shirokanedai Minato‐ku Tokyo 108‐8639 Japan; ^6^ Department of Surgery The University of Chicago 5841 S. Maryland Ave, MC2115 Chicago Illinois 60637

**Keywords:** HER2, human cancer, lysine methylation, SMYD3

## Abstract

HER2 is a receptor tyrosine kinase, which is amplified and overexpressed in a subset of human cancers including breast and gastric cancers, and is indicated in its involvement in progression of cancer. Although its specific ligand(s) has not been detected, HER2 homodimerization, which is critical for its activation, is considered to be dependent on its expression levels. Here, we demonstrate a significant role of HER2 methylation by protein lysine methyltransferase SMYD3 in HER2 homodimerization. We found that SMYD3 trimethylates HER2 protein at lysine 175. HER2 homodimerization was enhanced in the presence of SMYD3, and substitution of lysine 175 of HER2 with alanine (HER2‐K175A) reduced the formation of HER2 homodimers. Furthermore, HER2‐K175A revealed lower level of autophosphorylation than wild‐type HER2. We also identified that knockdown of SMYD3 attenuated this autophosphorylation in breast cancer cells. Our results imply that SMYD3‐mediated methylation of HER2 at Lysine 175 may regulate the formation of HER2 homodimer and subsequent autophosphorylation and suggest that the SMYD3‐mediated methylation pathway seems to be a good target for development of novel anti‐cancer therapy.

## Introduction

Human epidermal growth factor receptor 2 (EGFR2, also called as ERBB2 and HER2), a member of the epidermal growth factor receptor family of transmembrane receptor tyrosine kinases, is one of essential mediators of cell proliferation and differentiation in embryonic and adult tissues 1. Abnormal activation of HER2 is involved in development and progression of various types of cancer 2, 3; in particular, HER2 amplification is observed in 18–25% of human breast cancers 3, and is correlated with poor prognosis 4. This family protein is comprised of three main domains, extracellular domain (ECD), transmembrane domain (TM), and intracellular domain (ICD). EGFR family proteins except HER2 bind to specific ligands through their ECD, and cause structural change to their activated forms, and then interact with a partner protein 5, 6, 7. A HER2‐specific ligand(s) has not been identified 8, but HER2 protein is known to make a homodimer or a heterodimer with a member of other EGFR proteins, and then drive autophosphorylation in C‐terminal tyrosine residues, followed by activation of its downstream pathways 1, 9. Thus, its dimer formation is essential for initiating the signaling. It has been reported that HER2 homodimer is increased according to the increase in HER2 molecules on the cell surface 10. However, the regulatory mechanism of HER2 homodimerization is not fully understood.

SET and MYND domain‐containing protein 3 (SMYD3) is a protein lysine methyltransferase, and is overexpressed in a wide range of cancers, including breast, colorectal, hepatocellular, lung, and pancreatic carcinomas 11, 12, 13, 14, 15. Several lines of evidence have indicated that SMYD3 plays a pivotal role in human tumorigenesis through methylation of histone and nonhistone protein substrates 16, 17, 18, 19, 20, 21. In this study, we demonstrated trimethylation of a lysine 175 residue of HER2 by SMYD3, which may affect the HER2 homodimerization and the activation of its downstream pathways. Our findings may suggest that SMYD3 is likely to be an important target for development of a novel class of anti‐cancer drugs.

## Material and Methods

### Cell lines

293T, HeLa, MCF7, and ZR‐75‐1 cell lines were obtained from American Type Culture Collection (ATCC; Manassas, VA), and authentication was tested by DNA profiling for polymorphic short tandem repeat (STR) markers (Table S1). All cell lines were grown in monolayers in appropriate media supplemented with 10% fetal bovine serum and 1% antibiotic/antimycotic solution (Sigma‐Aldrich; St. Louis, MO): Dulbecco's modified Eagle's medium (D‐MEM) for 293T cells; Eagle's Minimum Essential Medium (E‐MEM) for HeLa and MCF7 cells; RPMI‐1640 medium for ZR‐75‐1 cells. Cells were transfected with FuGENE^®^ HD (Promega; Madison, WI) transfection reagent according to the manufacturer's recommendations 22.

### Antibodies

The following primary antibodies were used: anti‐FLAG (rabbit, F7425; Sigma‐Aldrich; dilution used in WB: 1:1000), anti‐HA (rabbit, Y‐11; Santa Cruz Biotechnology; Santa Cruz, CA; dilution used in ICC: 1:1000), anti‐SMYD3 (rabbit, D2Q4V; Cell Signaling Technology; Danvers, MA; dilution used in WB: 1:1000), anti‐HER2 (rabbit, 29D8; Cell Signaling Technology; dilution used in WB: 1:1000), anti‐phospho HER2 (Tyr 1248) (rabbit, #2247; Cell Signaling Technology; dilution used in WB: 1:500), anti‐EGFR (rabbit, D38B1; Cell Signaling Technology; dilution used in WB: 1:1000), anti‐ACTB (rabbit, #4967; Cell Signaling Technology; dilution used in WB: 1:1000), anti‐histone H3 (rabbit, ab1791; Abcam; Cambridge, UK; diluted used in: 1:1000), anti‐AKT (rabbit, C67E7; Cell Signaling Technology; dilution used in WB: 1:1000), anti‐phospho AKT (Ser 473) (mouse, 587F11; Cell Signaling Technology; dilution used in WB: 1:1000), anti‐PLCγ1 (rabbit, D9H10; Cell Signaling Technology; dilution used in WB: 1:1000), anti‐phospho PLCγ1 (Tyr 783) (rabbit, #2821; Cell Signaling Technology; dilution used in WB: 1:1000), anti‐p44/42 MAPK (Erk1/2) (rabbit, #9102; Cell Signaling Technology; dilution used in WB: 1:1000), anti‐phospho p44/42 MAPK (Erk1/2) (Thr202/Tyr204) (rabbit, D13.14.4E; Cell Signaling Technology; dilution used in WB: 1:1000).

### In vitro methyltransferase assay

Recombinant GST‐HER2 (H00002064‐P01, Novus biologicals, Littleton, CO) was incubated with SMYD3 enzyme and 2 *μ*Ci *S*‐adenosyl‐l‐[methyl‐3H]‐methionine (SAM; PerkinElmer, Branchburg, NJ) in a mixture of methylase activity buffer (50 mmol/L Tris‐HCl at pH 8.8, 10 mmol/L dithiothreitol (DTT), and 10 mmol/L MgCl_2_), for 3 h at 30°C. After denaturation, samples were subjected to SDS‐PAGE, and visualized by fluorography using EN^3^HANCE^™^ Spray Surface Autoradiography Enhancer (PerkinElmer). Loading proteins were visualized by MemCode^™^ Reversible Stain (Thermo Fisher Scientific, Waltham, MA).

### Mass spectrometry

The reaction samples of in vitro methyltransferase assay were subjected to SDS‐PAGE and stained with Simply Blue Safe Stain (Thermo Fisher Scientific). The bands corresponding to HER2 were excised and digested in gel with trypsin. Then the digested peptides were analyzed by nano liquid chromatography–tandem mass spectrometry (LC‐MS/MS) using Q Exactive mass spectrometer (Thermo Fisher Scientific). The peptides were separated using nano ESI spray column (75 *μ*m [ID] × 100 mm [L], NTCC analytical column C18, 3 *μ*m, Nikkyo Technos; Tokyo, Japan) with a linear gradient of 0–35% buffer B (100% acetonitrile and 0.1% formic acid) at a flow rate of 300 nL/min over 10 min (Easy nLC; Thermo Fisher Scientific). The mass spectrometer was operated in the positive‐ion mode, and the MS and MS/MS spectra were acquired with a data‐dependent TOP10 method. The MS/MS spectra were searched against the in‐house database using local MASCOT server (version 2.5; Matrix Sciences; Tokyo, Japan).

### Western Blot

Samples were prepared from the cells lysed with CelLytic^™^ M mammalian cell lysis reagent (Sigma‐Aldrich) containing a complete protease inhibitor cocktail (Roche Applied Science; Bavaria, Germany) and a phosphatase inhibitor cocktail (Roche Applied Science), and whole cell lysates or IP products were transferred to nitrocellulose membrane. Protein bands were detected by incubating with horseradish peroxidase‐conjugated antibodies (GE Healthcare; Buckinghamshire, UK) and visualizing with Enhanced Chemiluminescence (GE Healthcare).

### Immunoprecipitation

Transfected 293T and HeLa cells were lysed with CelLytic^™^ M supplemented with a complete protease inhibitor cocktail (Roche Applied Science) and a phosphatase inhibitor cocktail (Roche Applied Science). Cell extracts were incubated with anti‐FLAG^®^ M2 affinity gel or anti‐HA‐agarose overnight. After the beads were washed three times with PBS, proteins bound to the beads were eluted by elution buffer (3X FLAG^®^ peptide (Sigma‐Aldrich) or HA peptide (Sigma‐Aldrich) in PBS) containing a complete protease inhibitor cocktail (Roche Applied Science) and a phosphatase inhibitor cocktail (Roche Applied Science). Eluted samples were boiled with Lane Marker Sample Buffer (Thermo Fisher Scientific), and used for western blot analysis.

### siRNA transfection and cell growth assay

siRNA oligonucleotide duplexes were purchased from Sigma‐Aldrich for targeting the human *SMYD3* transcripts (SASI Hs01_00188121 and SASI Hs01_00188125). siNegative control (siNC), which consists of three different oligonucleotide duplexes, was used as a control siRNA (Cosmo Bio; Tokyo, Japan)^23^, ^24^. siRNA sequences are described in Table S2. siRNA duplexes (100 nmol/L final concentration) were transfected into ZR‐75‐1 and MCF7 cells with Lipofectamine^®^ RNAiMax Reagent (Thermo Fisher Scientific). After 96 h of incubation, cell extracts are fractionated into cytoplasmic protein and nuclear protein using NE‐PER Nuclear and Cytoplasmic Extraction Reagents (Thermo Fisher Scientific).

## Results

### SMYD3 methylates lysine residue in the ECD of HER2

**Figure 1:**
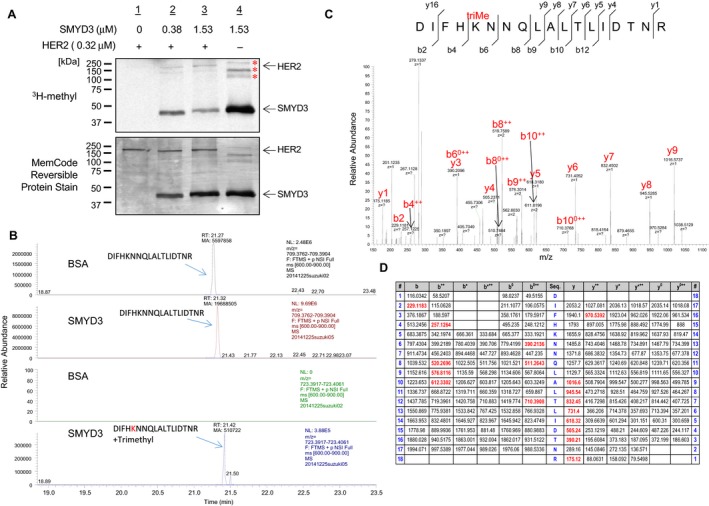
SMYD3 trimethylates HER2 at Lys 175. (A) In vitro methyltransferase assay of HER2. Recombinant GST‐HER2 protein was incubated with different concentration of SMYD3 in the presence of ^3^H‐SAM, and methylation signal was detected by autoradiography (upper panel). Amounts of loading proteins were evaluated by staining with MemCode^™^ Reversible Protein Stain (lower panel). *: nonspecific signals of SMYD3 automethylation. (B) MS chromatograms of unmodified and the trimethylated HER2 171‐188 peptide. (C) The MS‐MS spectrum corresponding to the trimethylated HER2 171‐188 peptide. The 42 Da increase of the Lys175 was observed. (D) LC‐MS/MS analysis showed trimethylation of Lys 175. Theoretical values of MS fragments are summarized.

To investigate whether HER2 could be a substrate of any protein methyltransferase(s), we first performed an in vitro methyltransferase assay using several protein methyltransferases for an initial screening, and found that SMYD3 possibly methylates HER2 protein. To validate this possibility, we further conducted an in vitro methyltransferase assay and observed dose‐dependent HER2 methylation by SMYD3 (Fig. 1A). To identify a methylation site(s) of HER2 mediated by SMYD3, we performed liquid chromatography–tandem mass spectrometry (LC‐MS/MS) analysis of in vitro‐methylated HER2 protein and identified that a lysine 175 (Lys 175) residue in the ECD of HER2 was trimethylated by SMYD3 (Fig. 1B and C). Lys 175 was previously suggested as an ubiquitination site, but was not well characterized including whether this site is monoubiquitinated or polyubiquitinated 25. To investigate the biological significance of this methylation, we performed an ubiquitination assay as well as cycloheximide (CHX)‐chase analysis using the methods reported previously 26, 27, but we were unable to confirm polyubiquitination at this residue or found no evidence indicating the importance of this methylation on the protein stability (data not shown).

### SMYD3‐mediated methylation at Lys 175 affects the phosphorylation level of HER2

**Figure 2:**
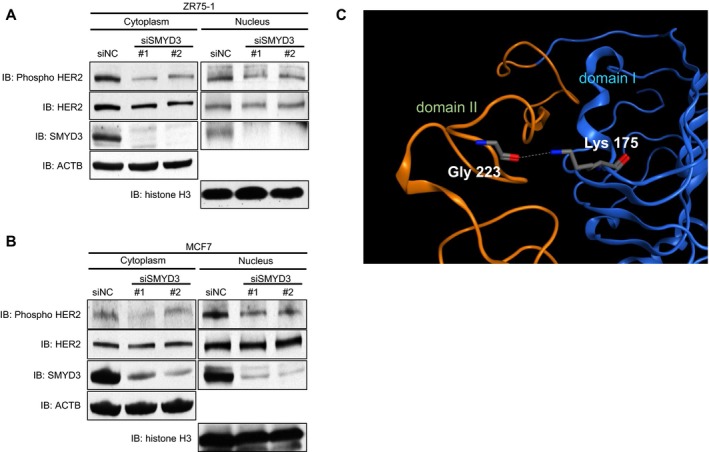
Knockdown of SMYD3 attenuates HER2 activity. (A and B) Effects of SMYD3 knockdown on HER2 phosphorylation levels in ZR‐75‐1 cells (A) and MCF7 cells (B). Cells were transfected with one control siRNA (siNC) or either of two SMYD3 siRNAs (#1 and #2). After incubation for 96 h, cell extracts were fractionated into cytoplasmic and nuclear proteins, then samples were immunoblotted with anti‐phospho HER2 (Tyr 1248) (#2247), anti‐HER2 (29D8), anti‐SMYD3 (D2Q4V), anti‐ACTB (#4967), and anti‐Histone H3 (ab1791). (C) The known three‐dimensional structure of the ECD of HER2. Only part of domain I (blue) and domain II (orange) is shown. The side‐chain amino group of Lys 175 in domain I makes a hydrogen bond with the backbone carbonyl group of Gly 223 in domain II. The drawing was prepared from the Protein Data Bank (entry code, 3WLW)^33^ using *Molecular Operating Environment* (MOE), 2015.10 (Chemical Computing Group Inc.).

We previously reported that molecular functions of lysine methylation are classified into at least five different classes including one class to regulate further modification(s) of a substrate protein 13. To examine whether SMYD3‐mediated methylation influences the phosphorylation status of HER2 protein, we knocked down SMYD3 in breast cancer cell lines using specific siRNAs and compared autophosphorylation levels of HER2 at Tyr 1248 that was indicated to be essential for HER2 activity 28. We found that siSMYD3 treatment clearly attenuated the phosphorylation level of HER2 in both ZR‐75‐1 and MCF7 cells (Fig. 2A and B).

To gain insight into possible effects of the methylation, we examined the known three‐dimensional structure of the ECD of HER2 (Fig. 2C). The ECD consists of four structural domains I, II, III, and IV. Of them, domain II is known to form the dimerization interface. Lys 175 is located in domain I, and its side‐chain amino group makes a hydrogen bond with the backbone carbonyl group of a glycine 223 (Gly 223) residue in domain II. The methylation of Lys 175 can disrupt the hydrogen bond. It is possible that the disruption may allow the domains to change their interdomain spatial relationship and then affect the dimerization event.

### SMYD3‐mediated Lys 175 methylation affects the formation of HER2 homodimer

**Figure 3:**
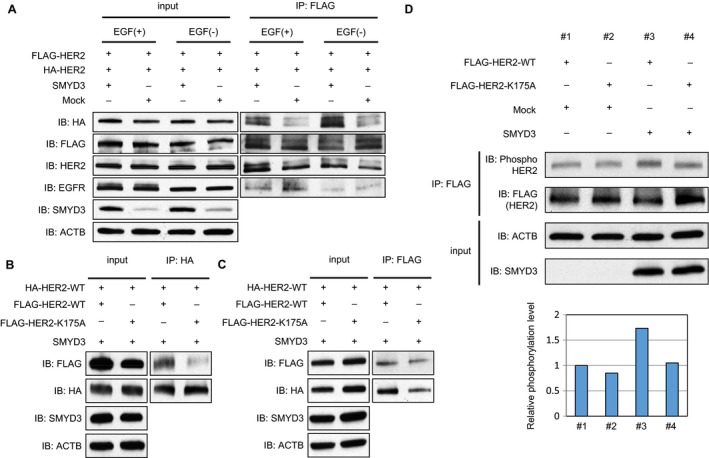
SMYD3‐mediated methylation enhances the formation of HER2 homodimer. (A) HeLa cells were transfected with FLAG‐HER2 and HA‐HER2, with Mock vector or SMYD3‐expressing vector. After 24 h of incubation, cells were treated with 0 or 100 ng/mL of EGF. Cell extracts were immunoprecipitated with anti‐FLAG ^®^ M2 affinity gel, and immunoblotted with anti‐HA (Y‐11), anti‐FLAG (F7425), anti‐HER2 (29D8), anti‐EGFR (D38B1), anti‐SMYD3 (D2Q4V), and anti‐ACTB (#4967). (B and C) 293T cells were transfected with HA‐HER2‐WT, and FLAG‐HER2‐WT or FLAG‐HER2‐K175A in the presence of SMYD3 expression vector and incubated for 48 h. Cell lysates were immunoprecipitated with anti‐HA‐agarose. (B) or anti‐FLAG ^®^ M2 affinity gel. (C), then immunoblotted with anti‐FLAG (F7425), anti‐HA (Y‐11), anti‐SMYD3 (D2Q4V), and anti‐ACTB (#4967). (D) 293T cells were cotransfected with FLAG‐HER2‐WT or FLAG‐HER2‐K175A, and Mock vector or SMYD3‐expressing vector. After 48 h of incubation, cell lysates were immunoprecipitated with anti‐FLAG ^®^ M2 affinity gel and immunoblotted with anti‐FLAG (F7425), anti‐phospho HER2 (Tyr 1248) (#2247), anti‐SMYD3 (D2Q4V), and anti‐ACTB (#4967). The signal intensities of phosphorylated HER2 were quantified, and normalized by each FLAG level.

To assess the effect of SMYD3‐mediated HER2 methylation on the formation of HER2 homodimer, we transfected both FLAG‐tagged HER2 (FLAG‐HER2) and HA‐tagged HER2 (HA‐HER2) together with mock vector or SMYD3‐expressing vector into HeLa cells, followed by immunoprecipitation using anti‐FLAG^®^ M2 affinity gel. Subsequent western blot analysis showed that coimmunoprecipitation of HA‐HER2 was significantly increased in the presence of SMYD3 regardless of EGF stimulation (Fig. 3A). In contrast, coimmunoprecipitation of EGFR was unchanged in either the absence or presence of SMYD3 overexpression, indicating that SMYD3 enhances the HER2 homodimerization but not heterodimerization with other EGFR family members. To further verify this possibility, we prepared a vector‐expressing FLAG‐tagged HER2 with a substitution of Lys 175 with an alanine residue (FLAG‐HER2‐K175A). We transfected HA‐tagged wild‐type HER2 (HA‐HER2‐WT) vector and SMYD3 expression vector into 293T cells together with FLAG‐tagged wild‐type HER2 (FLAG‐HER2‐WT) vector or FLAG‐HER2‐K175A vector. After immunoprecipitation with monoclonal anti‐HA‐agarose, we performed western blot analysis and found that the coimmunoprecipitated FLAG‐HER2‐K175A protein level was significantly lower than that of FLAG‐HER2‐WT, indicating that this methylation site is critically important for HER2 homodimerization (Fig. 3B). We also transfected same vectors into 293T cells, and reversely immunoprecipitated cell extracts with anti‐FLAG^®^ M2 affinity gel and obtained a similar result as Figure 3B (Fig. 3C).

Subsequently, to verify the biological significance of HER2‐K175 methylation with SMYD3, we transfected FLAG‐HER2‐WT vector or FLAG‐HER2‐K175A vector into 293T cells with SMYD3 vector or mock vector, and compared the autophosphorylation level of HER2. As shown in Figure 3D, the phosphorylation level of WT‐HER2 was clearly elevated under the SMYD3‐overexpression condition, suggesting that SMYD3‐mediated methylation at Lys 175 may affect the formation of the HER2 homodimer and autophosphorylation status of HER2.

### Effects of SMYD3‐mediated methylation on downstream pathways

**Figure 4:**
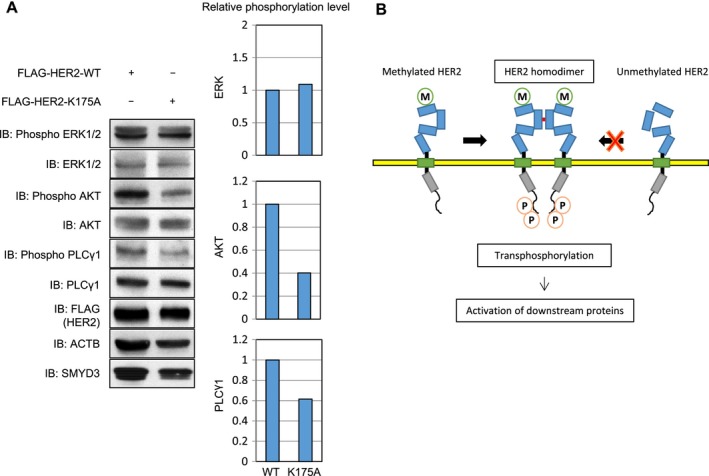
Effect of SMYD3‐mediated HER2 methylation on downstream pathways. (A) HeLa cells were transfected with FLAG‐HER2‐WT or FLAG‐HER2‐K175A, and incubated for 48 h. Cell lysates were immunoblotted with anti‐AKT (C67E7), anti‐phospho AKT (Ser 473) (587F11), anti‐PLCγ1 (D9H10, anti‐phospho PLCγ1 (Tyr 783) (#2821), anti‐ERK1/2 (#9102), anti‐phospho ERK1/2 (Thr202/Tyr204) (D13.14.4E), anti‐FLAG (F7425), anti‐SMYD3 (D2Q4V), and anti‐ACTB (#4967). (B) The schema of the effect of SMYD3‐mediated methylation on HER2 function.

Three growth signaling pathways, PI3K‐AKT, RAS‐MAPK, and PLCγ‐PKC pathways, are known to be mediated by HER2 activation. MAP3K2 was previously reported as a substrate of SMYD3 and the SMYD3‐mediated methylation was suggested to affect the phosphorylation status of ERK1/2 17. Since phosphorylation levels of these downstream genes are enhanced by HER2 overexpression 29, we introduced WT‐ or K175A‐ HER2 vector into HeLa cells and compared the phosphorylation levels of downstream genes. Expectedly, phosphorylation levels of AKT and PLCγ1 were much higher in the cells transfected with WT‐HER2 than those with K175A‐HER2 (Fig. 4A).

## Discussion

HER2 is well known to play an essential role in tumorigenesis in several types of cancer through activation of its downstream signaling pathways involved in cell proliferation, differentiation, angiogenesis, and apoptosis 3. Dimer formation is considered to be an essential process to activate these downstream signaling pathways. Since HER2‐specific ligand has never been identified, overexpression of HER2 is thought as the only mechanism to regulate homodimerization 10.

In this study, we have demonstrated that HER2 was trimethylated at Lys 175 by SMYD3, and that SMYD3‐mediated HER2 methylation enhanced HER2 homodimerization and HER2‐downstream pathways. In addition, we showed that knockdown of SMYD3 reduced the HER2 phosphorylation level and concordantly overexpression of SMYD3 increased its phosphorylation level, indicating that SMYD3‐induced HER2 methylation is likely to enhance HER2 phosphorylation. The structural analysis implied that Lys 175 on domain I makes a hydrogen bond with Gly 223 on domain II and SMYD3‐mediated methylation at Lys 175 could disrupt the hydrogen bond. Hence, the interdomain interaction between domain I and domain II might be influenced and then the formation of HER2 dimer could be enhanced. Indeed, we confirmed that the interaction of HA‐HER2 and FLAG‐HER2 was significantly higher in the presence of SMYD3. In addition, HER2‐K175A protein, in which a methylation lysine site in HER2 was substituted with an alanine residue, showed a very low interaction with HER2‐WT protein, compared to the interaction between HA‐HER2‐WT and FLAG‐HER2‐WT, indicating that SMYD3 may play a pivotal role on HER2 activation through enhancement of HER2 homodimerization (Fig. 4C). Moreover, the other important finding of this study is that the methylation site of HER2 is located in the ECD. Indeed, ECDs in some chemokine receptors are also posttranslationally modified. For instance, human chemokine receptors CCR2b, CCR5, CX_3_CR1, and CXCR4 are reported to be sulfated and/or glycosylated at the ECDs 30, 31, 32. These modifications seem to have diverse consequences for receptor ligand‐binding activities, which may affect their functions as coreceptors of the human immunodeficiency virus infection. In addition, current bioinformatics analysis and subcellular localization analysis using high‐quality antibody indicate that SMYD3 appears to be localized into the Golgi apparatus beside the nucleus and cytoplasm, implying that the ECD of HER2 may be methylated in the Golgi apparatus by SMYD3. Although the diverse functions of posttranslational modifications at ECDs of transmembrane receptor tyrosine kinases such as the epidermal growth factor receptor family still remain to be elucidated, further studies may unveil their physiological importance besides our current findings.

As mentioned above, we and other groups reported that SMYD3 was highly expressed in various types of human cancer 11, 12, 13, 14, 15, and is implicated to have an oncogenic function 18, 19, 20, 21. However, the biological significance of nonhistone protein methylation by SMYD3 has not been well characterized. In recent years, VEGFR1 and MAP3K2 were reported as substrates of SMYD3 and the possible functions of methylation on these proteins were discussed 16, 17. These findings imply that the protein lysine methyltransferase SMYD3 is thought to have unique methylation functions that influence known signaling pathways.

In summary, we have demonstrated that SMYD3 may play its oncogenic role through HER2 methylation. This study is the first report indicating the high correlation between SMYD3‐mediated methylation and HER2 homodimerization, supporting that the development of specific inhibitors targeting SMYD3 methylation pathway will be a promising approach for development of a novel class of anti‐cancer therapy.

## Conflicts of Interest

Y. Nakamura is a stock holder and a scientific advisor of OncoTherapy Science, Inc, and Y. Matsuo is an employee of OncoTherapy Science, Inc. There are no potential conflicts of interest by the other authors.

## Supporting information


**Table S1.** Information of certificated cell lines.
**Table S2.** siRNA sequences.

## References

[1] Olayioye, M. A. , R. M. Neve , H. A. Lane , and N. E. Hynes . 2000 The ErbB signaling network: receptor heterodimerization in development and cancer. EMBO J. 19:3159–3167. 10880430 10.1093/emboj/19.13.3159 PMC313958

[2] Slamon, D. J. , G. M. Clark , S. G. Wong , W. J. Levin , A. Ullrich , and W. L. McGuire . 1987 Human breast cancer: correlation of relapse and survival with amplification of the HER‐2/neu oncogene. Science 235:177–182. 3798106 10.1126/science.3798106

[3] Baselga, J. , and S. M. Swain . 2009 Novel anticancer targets: revisiting ERBB2 and discovering ERBB3. Nat. Rev. Cancer 9:463–475. 19536107 10.1038/nrc2656

[4] Rubin, I. , and Y. Yarden . 2001 The basic biology of HER2. Ann. Oncol. 12(Suppl. 1):S3–S8. 10.1093/annonc/12.suppl_1.s3 11521719

[5] Arteaga, C. L. , and J. A. Engelman . 2014 ERBB receptors: from oncogene discovery to basic science to mechanism‐based cancer therapeutics. Cancer Cell 25:282–303. 24651011 10.1016/j.ccr.2014.02.025 PMC4018830

[6] Iqbal, N. , and N. Iqbal . 2014 Human epidermal growth factor receptor 2 (HER2) in cancers: overexpression and Therapeutic Implications. Mol. Biol. Int. 2014:852748. 25276427 10.1155/2014/852748 PMC4170925

[7] Garrett, T. P. , N. M. McKern , M. Lou , et al. 2003 The crystal structure of a truncated ErbB2 ectodomain reveals an active conformation, poised to interact with other ErbB receptors. Mol. Cell 11:495–505. 12620236 10.1016/s1097-2765(03)00048-0

[8] Hynes, N. E. , and H. A. Lane . 2005 ERBB receptors and cancer: the compllexity of targeted inhibitors. Nat. Rev. Cancer 5:341–354. 15864276 10.1038/nrc1609

[9] Ferguson, K. M. , M. B. Berger , J. M. Mendrola , H. S. Cho , D. J. Leahy , and M. A. Lemmon . 2003 EGF activates its receptor by removing interactions that autoinhibit ectodomain dimerization. Mol. Cell 11:507–517. 12620237 10.1016/s1097-2765(03)00047-9

[10] Yarden, Y. , and M. X. Sliwkowski . 2001 Untangling the ErbB signalling network. Nat. Rev. Mol. Cell Biol. 2:127–137. 11252954 10.1038/35052073

[11] Hamamoto, R. , Y. Furukawa , M. Morita , et al. 2004 SMYD3 encodes a histone methyltransferase involved in the proliferation of cancer cells. Nat. Cell Biol. 6:731–740. 15235609 10.1038/ncb1151

[12] Hamamoto, R. , F. P. Silva , M. Tsuge , et al. 2006 Enhanced SMYD3 expression is essential for the growth of breast cancer cells. Cancer Sci. 97:113–118. 16441421 10.1111/j.1349-7006.2006.00146.x PMC11159510

[13] Hamamoto, R. , V. Saloura , and Y. Nakamura . 2015 Critical roles of non‐histone protein lysine methylation in human tumorigenesis. Nat. Rev. Cancer 15:110–124. 25614009 10.1038/nrc3884

[14] Tsuge, M. , R. Hamamoto , F. P. Silva , et al. 2005 A variable number of tandem repeats polymorphism in an E2F‐1 binding element in the 5’ flanking region of SMYD3 is a risk factor for human cancers. Nat. Genet. 37:1104–1107. 16155568 10.1038/ng1638

[15] Silva, F. P. , R. Hamamoto , M. Kunizaki , M. Tsuge , Y. Nakamura , and Y. Furukawa . 2008 Enhanced methyltransferase activity of SMYD3 by the cleavage of its N‐terminal region in human cancer cells. Oncogene 27:2686–2692. 17998933 10.1038/sj.onc.1210929

[16] Kunizaki, M. , R. Hamamoto , F. P. Silva , et al. 2007 The lysine 831 of vascular endothelial growth factor receptor 1 is a novel target of methylation by SMYD3. Can. Res. 67:10759–10765. 10.1158/0008-5472.CAN-07-1132 18006819

[17] Mazur, P. K. , N. Reynoird , P. Khatri , et al. 2014 SMYD3 links lysine methylation of MAP3K2 to Ras‐driven cancer. Nature 510:283–287. 24847881 10.1038/nature13320 PMC4122675

[18] Liu, Y. , X. Luo , J. Deng , Y. Pan , L. Zhang , and H. Liang . 2015 SMYD3 overexpression was a risk factor in the biological behavior and prognosis of gastric carcinoma. Tumour Biol. 36:2685–2694. 25472580 10.1007/s13277-014-2891-z

[19] Peserico, A. , A. Germani , P. Sanese , et al. 2015 A SMYD3 small‐molecule inhibitor impairing cancer cell growth. J. Cell. Physiol. 230:2447–2460. 25728514 10.1002/jcp.24975 PMC4988495

[20] Shen, B. , M. Tan , X. Mu , et al. 2016 Upregulated SMYD3 promotes bladder cancer progression by targeting BCLAF1 and activating autophagy. Tumour Biol. 37:7371–7381. 26676636 10.1007/s13277-015-4410-2

[21] Sarris, M. E. , P. Moulos , A. Haroniti , A. Giakountis , and I. Talianidis . 2016 Smyd3 is a transcriptional potentiator of multiple cancer‐promoting genes and required for liver and colon cancer development. Cancer Cell 29:354–366. 26908355 10.1016/j.ccell.2016.01.013

[22] Sone, K. , L. Piao , M. Nakakido , et al. 2014 Critical role of lysine 134 methylation on histone H2AX for gamma‐H2AX production and DNA repair. Nat. Commun. 5:5691. 25487737 10.1038/ncomms6691 PMC4268694

[23] Yoshimatsu, M. , G. Toyokawa , S. Hayami , et al. 2011 Dysregulation of PRMT1 and PRMT6, Type I arginine methyltransferases, is involved in various types of human cancers. Int. J. Cancer 128:562–573. 20473859 10.1002/ijc.25366

[24] Deng, X. , G. Von Keudell , T. Suzuki , et al. 2015 PRMT1 promotes mitosis of cancer cells through arginine methylation of INCENP. Oncotarget. 6:35173–35182. 26460953 10.18632/oncotarget.6050 PMC4742097

[25] Anania, V. G. , V. C. Pham , X. Huang , A. Masselot , J. R. Lill , and D. S. Kirkpatrick . 2014 Peptide level immunoaffinity enrichment enhances ubiquitination site identification on individual proteins. Mol. Cell Proteomics 13:145–156. 24142993 10.1074/mcp.M113.031062 PMC3879610

[26] Takawa, M. , H. S. Cho , S. Hayami , et al. 2012 Histone lysine methyltransferase SETD8 promotes carcinogenesis by deregulating PCNA expression. Can. Res. 72:3217–3227. 10.1158/0008-5472.CAN-11-3701 22556262

[27] Nunes, J. , H. Zhang , N. Angelopoulos , et al. 2016 ATG9A loss confers resistance to trastuzumab via c‐Cbl mediated Her2 degradation. Oncotarget 7:27599–27612. 27050377 10.18632/oncotarget.8504 PMC5053674

[28] DiGiovanna, M. P. , M. A. Lerman , R. J. Coffey , W. J. Muller , R. D. Cardiff , and D. F. Stern . 1998 Active signaling by Neu in transgenic mice. Oncogene 17:1877–1884. 9778054 10.1038/sj.onc.1202091

[29] Hartman, Z. , H. Zhao , and Y. M. Agazie . 2013 HER2 stabilizes EGFR and itself by altering autophosphorylation patterns in a manner that overcomes regulatory mechanisms and promotes proliferative and transformation signaling. Oncogene 32:4169–4180. 23027125 10.1038/onc.2012.418 PMC3538112

[30] Gutierrez, J. , L. Kremer , A. Zaballos , I. Goya , A. C. Martinez , and G. Marquez . 2004 Analysis of post‐translational CCR8 modifications and their influence on receptor activity. J. Biol. Chem. 279:14726–14733. 14736884 10.1074/jbc.M309689200

[31] Preobrazhensky, A. A. , S. Dragan , T. Kawano , et al. 2000 Monocyte chemotactic protein‐1 receptor CCR2B is a glycoprotein that has tyrosine sulfation in a conserved extracellular N‐terminal region. J. Immunol. 165:5295–5303. 11046064 10.4049/jimmunol.165.9.5295

[32] Farzan, M. , T. Mirzabekov , P. Kolchinsky , et al. 1999 Tyrosine sulfation of the amino terminus of CCR5 facilitates HIV‐1 entry. Cell 96:667–676. 10089882 10.1016/s0092-8674(00)80577-2

[33] Hu, S. , Y. Sun , Y. Meng , et al. 2015 Molecular architecture of the ErbB2 extracellular domain homodimer. Oncotarget 6:1695–1706. 25633808 10.18632/oncotarget.2713 PMC4359325

