# Supplementary material for: Protein lysine methyltransferase SMYD3 is involved in tumorigenesis through regulation of HER2 homodimerization
Source: Cancer Med. 2017 Jun 22;6(7):1665–72. doi: 10.1002/cam4.1099 (PMC5504314; doi:10.1002/cam4.1099)
Supplement: Supplementary file 1 — Table S1. Information of certificated cell lines. Table S2. siRNA sequences. [file CAM4-6-1665-s001.docx]

**Supporting information**

**The protein lysine methyltransferase SMYD3 is involved in tumorigenesis through regulation of HER2 homodimerization**

The file contains

Supplementary Tables S1 - S2

**Supplementary Table S1: Information of certificated cell lines**

| **Name** | **Origin** | **Certification institution** | **Tested method** | **DNA profile or characteristics** |
| --- | --- | --- | --- | --- |
| MCF7 | human breast cancer | ATCC | STR | Amelogenin: X/X CSF1PO: 10/10 D13S317: 11/11 D16S539: 11/12 D5S818: 12/12 D7S820: 8/9 THO1: 6/6 TPOX: 9/12 vWA: 14/15 D3S1358: 16/16 D21S11: 30/30 D18S51: 14/14 Penta E: 7/12 Penta D: 12/12 D8S1179: 10/14 FGA: 23/25 |
| ZR-75-1 | human breast cancer | ATCC | STR | Amelogenin: X CSF1PO: 10,11 D13S317: 9 D16S539: 11 D5S818: 13 D7S820: 10,11 THO1: 7,9.3 TPOX: 8 vWA: 16,18 |
| 293T | human embryonic kidney fibroblast | ATCC | STR | Amelogenin: X CSF1PO: 11, 12 D13S317: 12, 14 D16S539: 9, 13 D5S818: 8, 9 D7S820: 11 THO1: 7, 9.3 TPOX: 11 vWA: 16, 18, 19 |
| HeLa | human cervix carcinoma | ATCC | STR | Amelogenin: X,Y CSF1PO: 11,12 D13S317: 11,14 D16S539: 9,11 D5S818: 11,12 D7S820: 10,11 THO1: 8 TPOX: 8 vWA: 15 |
| ATCC; American Type Culture Collection | | |  |  |
|  | | | |  |
|  | | | |  |

**Supplementary Table 2: siRNA sequences**

| **siRNA name** |  | **Sequence** |
| --- | --- | --- |
|  | Target#1 | Sense: 5’ AUCCGCGCGAUAGUACGUA 3’ |
| siNegative control |  | Antisense: 5’ UACGUACUAUCGCGCGGAU 3’ |
| (Cocktail) | Target#2 | Sense: 5’ UUACGCGUAGCGUAAUACG 3’ |
|  |  | Antisense: 5’ CGUAUUACGCUACGCGUAA 3’ |
|  | Target#3 | Sense: 5’ UAUUCGCGCGUAUAGCGGU 3’ |
|  |  | Antisense: 5’ ACCGCUAUACGCGCGAAUA 3’ |
| siSMYD3#1 |  | Sense: 5’ GAUUGAAGAUUUGAUUCUA 3’ |
|  |  | Antisense: 5’ UAGAAUCAAAUCUUCAAUC 3’ |
| siSMYD3#2 |  | Sense: 5’ CAGCAAUUCUGAACGGCUU 3’ |
|  |  | Antisense: 5’ AAGCCGUUCAGAAUUGCUG 3’ |
